# Ab initio calculations on structure and stability of BN/CC isosterism in azulene

**DOI:** 10.1038/s41598-023-37047-7

**Published:** 2023-06-24

**Authors:** Mohamed A. Abdel-Rahman, Kamal A. Soliman, Safwat Abdel-Azeim, Ahmed M. El-Nahas, Tetsuya Taketsugu, Takahito Nakajima, Asmaa B. El-Meligy

**Affiliations:** 1 Chemistry Department, Faculty of Science, Suez University, Suez, 43518 Egypt; 2 Chemistry Department, Faculty of Science, Benha University, Benha, 13518 Egypt; 3 Center for Integrative Petroleum Research (CIPR), College of Petroleum Engineering and Geosciences, King Fahd University of Petroleum and Minerals (KFUPM), Dhahran, 31261 Saudi Arabia; 4 Chemistry Department, Faculty of Science, Menoufia University, Shebin El-Kom, 32512 Egypt; 5 Department of Chemistry, Faculty of Science, Hokkaido University, Sapporo, 060-0810 Japan; 6 Institute for Chemical Reaction Design and Discovery (WPI-ICReDD), Hokkaido University, Sapporo, 060-0810 Japan; 7 Center for Computational Science, RIKEN, 7-1-26 Minatojima-Minami, Chuo, Kobe, 650-0047 Japan

**Keywords:** Chemistry, Theoretical chemistry

## Abstract

Herein, we investigated the thermodynamic stability and opto-electronic properties of a newly BN-doped azulene. The gas-phase formation enthalpies of 11 BN-doped azulene were calculated by the atomization energy method using three computational models (CBS-APNO, CBS-QB3, and G3MP2). The results suggest that AZ-1N9B exhibits the highest stability among the studied isomers. On the other hand, AZ-1B9N and AZ-9B10N display nearly equal stability with relative energies of 19.36 and 19.82 kcal/mol at CBS-QB3, respectively. These two isomers are considered the least stable among the investigated compounds. The frontier molecular orbitals (FMO), ionization energies (IE), and electron affinities (EA) of these isomers were discussed. Additionally, the electronic absorption spectra of the BN-doped azulenes were computed using the TD-B3LYP/6–31 + G(d,p) and TD-CAM-B3LYP level of theories, which using a long-range corrected hybrid functional in acetone. The computational results obtained in this research are align closely with the existing literature, thereby reinforcing the credibility and reliability of our findings.

## Introduction

The presence of isosterism, where compounds with the same number of atoms and electrons, can lead to the formation of unique heterocylic aromatic hydrocarbons. One example of such isosterism is the BN/CC isosterism, where BN bond replaces the CC bond^1,2^. The atomic displacement can lead to a change in electronic, optical and reactivity properties relative to their parent hydrocarbons^1–6^. This phenomenon has been used in drug design to improve the pharmacokinetics and pharmacodynamics of drugs^7^. It has also been used in materials science to enhance materials' chemical and physical properties for potential use in specific applications^1,2^. A dopant atom, such as boron and nitrogen, allows for the tunability of electronic and structural properties^8–10^.

Dewar et al. in 1958 reported a single CC/BN substitution in an aromatic compound^11^ and synthesized a good library of mono-^12^ and polycyclic^13^ compounds Recently, Liu et al.^14^ synthesized the least stable isomer of BN-doped naphthalene for optoelectronic properties and supported their findings with quantum chemical calculations. However, the slow progress in this area can be attributed to the lack of suitable synthetic methods^15^.

Azulene is an aromatic non-alternant isomer of naphthalene, consisting of a pentagonal and a heptagonal fused ring. Since its discovery, it has attracted significant interest. Azulene has unique properties like a dipole moment of 1.08 Debye^16^ compared to zero dipole moment for naphthalene. It also exhibits a narrow highest occupied molecular orbital (HOMO) –lowest unoccupied molecular orbital (LUMO) energy gap resulting from the non-mirror-symmetric orbitals^17^. Azulene derivatives have been used in the development of new advanced materials, including anion receptors/sensors^18^, molecular switches^19^, electrochromic materials^20^, conductive charge-transfer complexes^21^, liquid crystals^22^, organic/polymeric conductors^23^, and near-infrared (NIR) resonance materials^24^.

To the best of our knowledge, this work represents the first attempt to study a novel class of BN-doped aromatics, namely BN-doped azulene, compared to the recently reported experimental investigation of BN-doped naphthalene analogs^14^. The gas-phase enthalpies of formation for eleven isomers of BN-substituted azulene were computed using three ab initio composite methods, G3MP2, CBS-QB3, and CBS-APNO. In addition, naphthalene and its six BN-doped species were calculated for comparison. Furthermore, the electronic absorption ultraviolet–visible (UV–Vis) spectra in the gas phase and in acetone were calculated using the time-dependent density functional theory (TD-DFT)^25^. The frontier molecular orbital energies, ionization energies, electron affinities, and the density of states (DOS) have also been discussed.

## Computational details

All calculations were carried out using Gaussian 16 program^26^ using the multi-level ab initio composite methods G3MP2, CBS-QB3, and CBS-APNO developed for accurate thermochemical data^27–31^. Visualization of different structures was performed by chimeraX software^32^, while DOS is calculated using the GaussSum 3.0 program^33^.

Geometry optimization and frequency calculations at CBS-QB3 were performed with DFT/B3LYP^34,35^ using 6–311G(d,p) basis sets. Then, single-point energy calculations were performed at CCSD(T), MP4SDQ, and MP2 with different basis sets. Finally, CBS extrapolation is employed to get the final energy. However, CBS-APNO starts with geometry and frequency calculations at the HF/6–311G(d,p) level, followed by further optimization at the QCISD/6–311G(d,p). This high-level optimization step is used for four single-point energy calculations at the QCISD(T), MP2(full), HF, and MP2 levels. The G3MP2 combined method falls between CBS-QB3 and CBS-APNO regarding computational cost. It begins with geometry optimization at MP2(full)/6–31G(d) and zero-point vibrational energy from HF/6−31G(d). Similar to other composite methods, this optimization process is followed by a series of single-point energy calculations with QCISD(T)/6–31G(d,p), the most time-demanding step, followed by some corrections through basis set extensions at MP2 which eliminate the costly MP4 process in the G2MP2 method. The gas phase enthalpy of formation was computed using the atomization energy approach described by Ochterski^36^. The adiabatic electron affinity and ionization potentials for the considered systems were calculated from G3MP2 energies. Enthalpies of formation can be estimated from Eq. (1):

1 $$\Delta {H}_{\mathrm{f}}^{^\circ }\mathrm{gas}\left(\mathrm{S}\right)={E}_{e}\left(\mathrm{S}\right)+\mathrm{ZPVE}\left(\mathrm{S}\right)+\left[{H}_{298}\left(\mathrm{S}\right)-{H}_{0}\left(\mathrm{S}\right)\right]-\sum_{\mathrm{i}}^{\mathrm{atoms}}\{{E}_{\mathrm{e}}\left({\mathrm{X}}_{\mathrm{i}}\right)+[{H}_{298}\left({\mathrm{X}}_{\mathrm{i}}\right)-{ H}_{0}\left({\mathrm{X}}_{\mathrm{i}}\right)]\}+\sum_{\mathrm{i}}^{\mathrm{atoms}}\Delta {H}_{\mathrm{f}}^{^\circ }\mathrm{gas}({\mathrm{X}}_{\mathrm{i}}),$$

where *E*_e_(S) and *E*_e_(X_i_) are the calculated energy of molecule S and ith atom X, respectively. [*H*_298_(S) – *H*_0_(S)] and [*H*_298_ (X_i_) – *H*_0_(X_i_)] are the thermal corrections to the enthalpy for the molecule S and the individual atoms X, respectively. ZPVE is the zero-point vibrational energy of molecule S. The separated atomic enthalpies Δ*H*^°^_f_(X_i_) can be obtained from the NIST WebBook^37^. Ionization energy^38,39^ was calculated from Eq. (2):

2 $${\text{IE }} = E\left( {{\text{N}}^{ + } } \right) \, - E\left( {\text{N}} \right)$$

where *E* (N^+^) and *E* (N) are zero-point corrected energies of the molecular cation and the neutral molecule, respectively, at the same level of theory. Electron affinity (EA) ^39^ was estimated from Eq. (3):

3 $${\text{EA }} = E\left( {\text{N}} \right) \, - E\left( {{\text{N}}^{ - } } \right)$$

where *E* (N^-^) is the zero-point corrected energy of the molecular anion. Time-dependent density functional theory with the B3LYP functional (TD-B3LYP/6–31 + G(d,p)) and with the long-range corrected hybrid Coulomb attenuating method CAM-B3LYP^40^ (TD-CAM-B3LYP/6–31 + G (d,p)) were used to compute electronic absorption spectra of the considered compounds in acetone utilizing non-equilibrium polarizable continuum solvation model (PCM) at the B3LYP/6–31 + G (d,p) optimized gas phase geometry. HUMOs, LUMOs distribution and ESP were sketched using Gaussview^41^, while the ultraviolet–visible (UV–Vis) spectra were simulated using GaussSum 3.0 program^33^.

## Results and discussion

We have started our investigation by doing a benchmark calculation on the parent molecules (naphthalene and azulene). Our calculations of the zero-point corrected enthalpy of formations predicted that naphthalene is more stable than azulene, which can be attributed to the resonance stabilization energy. The calculated relative enthalpy of formation is 33.08 kcal/mol agrees well with the reported experimental value of 32.93 kcal/mol based on solid-state measurements^42^, with a deviation of only 0.15 kcal/mol. For naphthalene, the average experimental value was reported^43^ to be 35.98 ± 0.36 kcal/mol, as given in Table 1. This value is 3.58 and 5 kcal/mol higher than the values calculated by G3MP2 and CBS-APNO methods, respectively. However, the CBS-QB3 value lies at the upper limit of the experimental value.

**Table 1:** Enthalpy of formation (kcal/mol) for naphthalene, BN-doped naphthalene, azulene and BN-doped azulene.

Structures\Method	G3MP2	G3MP2[6]	Difference	CBS-APNO	CBS-QB3	Experiment
Napth	32.25	–	–	30.83	37.24	35.98 ± 0.36^43^
Napth-1B2N	13.64	13.00	0.64	10.04	16.89	–
Napth-1N2B	13.23	12.60	0.63	9.40	16.49	–
Napth-1N9B	22.34	21.70	0.64	19.76	26.43	–
Napth-2B3N	24.39	23.80	0.59	21.25	28.00	–
Napth-9N10B	26.12	25.50	0.62	23.71	29.98	–
Napth-1B9N	31.88	31.30	0.58	28.20	35.28	–
AZ	66.12	–	–	64.38	71.95	66.89^45^, 73.58 ^44^
AZ-1N2B	44.48	–	–	41.36	48.16	–
AZ-1B2N	53.44	–	–	50.94	57.50	–
AZ-1N9B	40.33	–	–	38.13	44.52	–
AZ-1B9N	59.71	–	–	57.39	63.88	–
AZ-6N7B	51.17	–	–	48.90	55.31	–
AZ-6B7N	47.12	–	–	44.60	51.03	–
AZ-7N8B	46.66	–	–	44.14	50.62	–
AZ-7B8N	53.51	–	–	–	57.67	–
AZ-8N9B	53.95	–	–	52.58	58.86	–
AZ-8B9N	44.96	–	–	41.80	48.26	–
AZ-9B10N	60.96	–	–	57.96	64.34	–

The gas-phase formation enthalpy of azulene at standard conditions was determined experimentally by Roth et al.^44^ and Kovats et al.^45^ as 73.61 and 66.92 kcal/mol, respectively. The calculated values at G3MP2 and CBS-QB3 levels agree with the experimental counterparts (Table 1). Although there is no experimental data for the BN-doped azulenes investigated in this study, we can discuss their stabilities with some confidence based on the above benchmark performed on naphthalene and azulene. Further, the reliability of the three methods employed in our study is well known to reproduce high accurate thermodynamic data.

Here, we studied the effect of different vicinal doping positions of BN on the enthalpy of the formation of azulene. Table 1 lists the calculated enthalpies of formation (kcal/mol) for 11 BN-doped azulene and 6 BN-doped naphthalene obtained from the atomization energy method using the G3MP2, CBS-QB3, and CBS-APNO methods.

**Figure 1:**
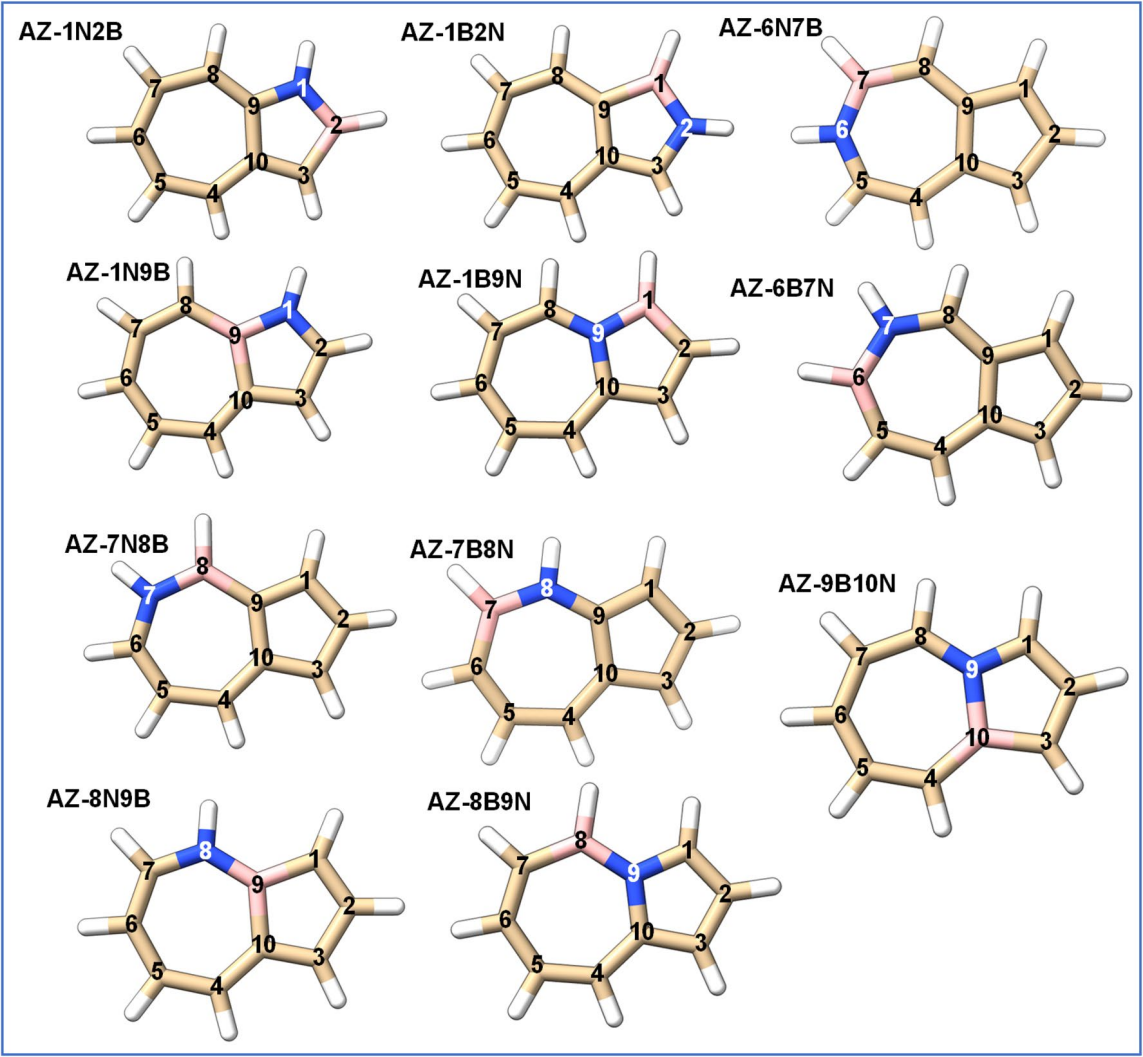
Optimized structures of BN-doped azulene isomers at B3LYP/6–311G(d,p) (optimization level of CBS-QB3 method) performed by chimeraX software (version 1)^32^.

**Figure 2:**
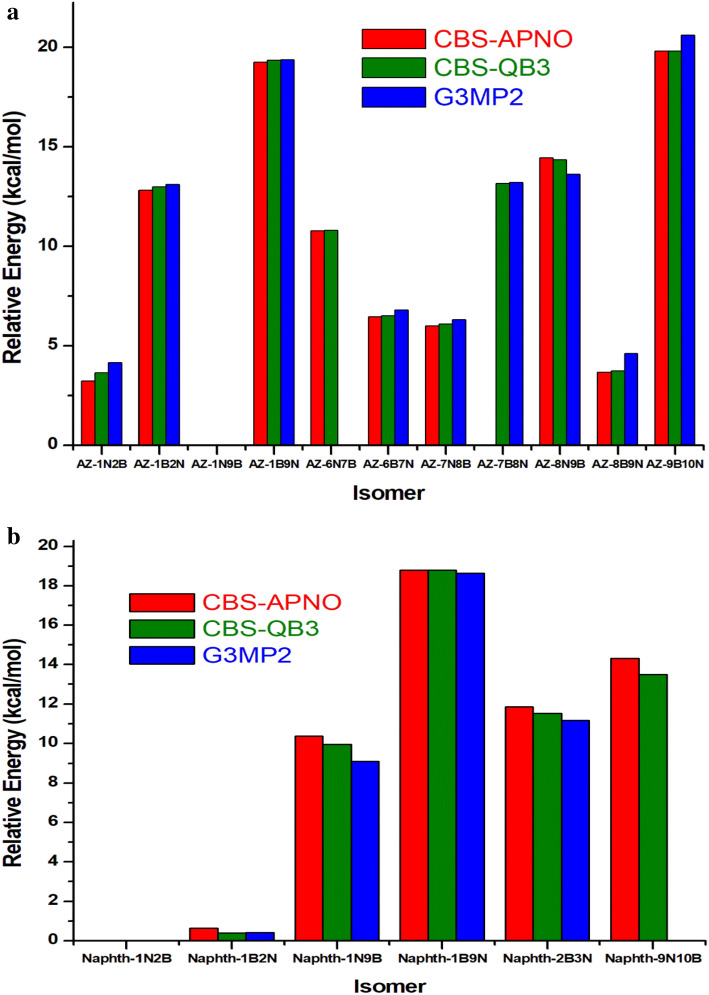
(**a**) Zero-point corrected relative energies of 11 BN-doped azulene isomers (kcal/mol) at CBS-APNO, CBS-QB3, and G3MP2 methods. (**b**) Zero-point corrected relative energies of 6 BN-doped naphthalene isomers (kcal/mol) at CBS-APNO, CBS-QB3, and G3MP2 methods.

The optimized structures of 11 BN-doped azulene isomers at B3LYP/6−311G(d,p) (optimization level of CBS-QB3 method) are depicted in Fig. 1, and the associated coordinates are reported in Table S1 in the supplementary information (SI). Figure 2 sketches the relative energies of the 11 BN-doped azulene isomers referenced to the most stable isomer AZ-1N9B using the three composite methods CBS-APNO, CBS-QB3, and G3MP2. These relative energies are also reported in Table S2. Figure 2 shows that AZ-1N9B is the most stable isomer, followed by AZ-1N2B and AZ-8B9N isomers with almost the same energies (relative energies of 3.64 and 3.74 kcal/mol), respectively. The least stable isomers are AZ-1B9N and AZ-9B10N with relative energies of 19.36 and 19.82 kcal/mol, respectively, at the CBS-QB3 method.

This may be attributed to the orbital amplitude being mostly concentrated at positions 1 and 3 of the azulene, favoring atoms with higher electronegativity at these positions. However, positions 2, 4, 6, and 8 have smaller orbital amplitudes and represent positions that would prefer nucleophilic substitution. Therefore, isomers with N substitutions at positions 1 or 3 are more stable than isomers with N at positions 2, 6, and 8. On the contrary, isostere with B at positions 2, 6, and 8 are more stable than isomers with B at positions 1 and 7. As shown in Table 1, the calculated formation enthalpies using G3MP2 and CBS-QB3 are close to those of the more expensive CBS-APNO method. Therefore, the discussion will be limited to the former two methods.

**Figure 3:**
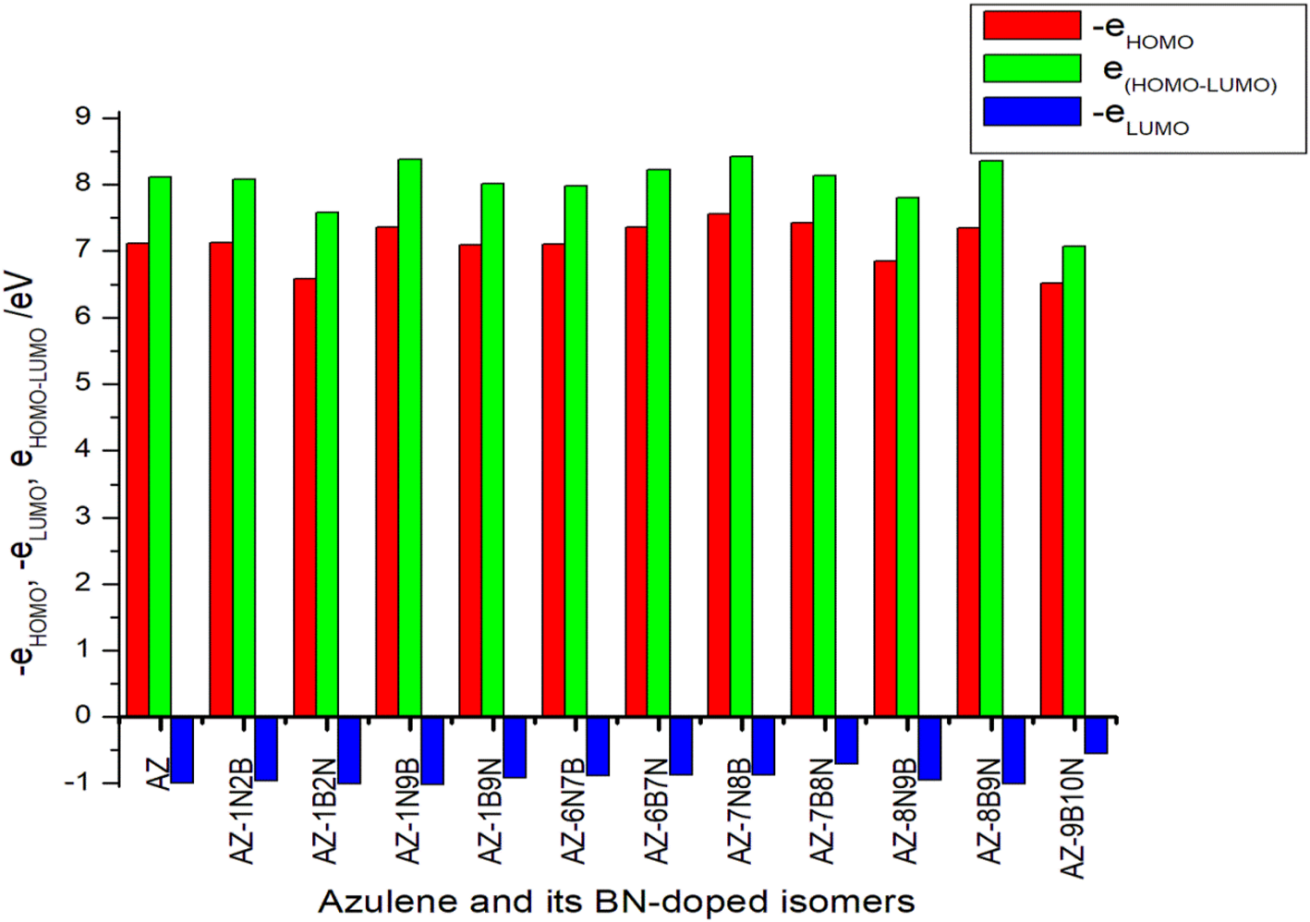
Orbital energies of azulene and 11 BN-doped isomers at G3MP2.

**Figure 4:**
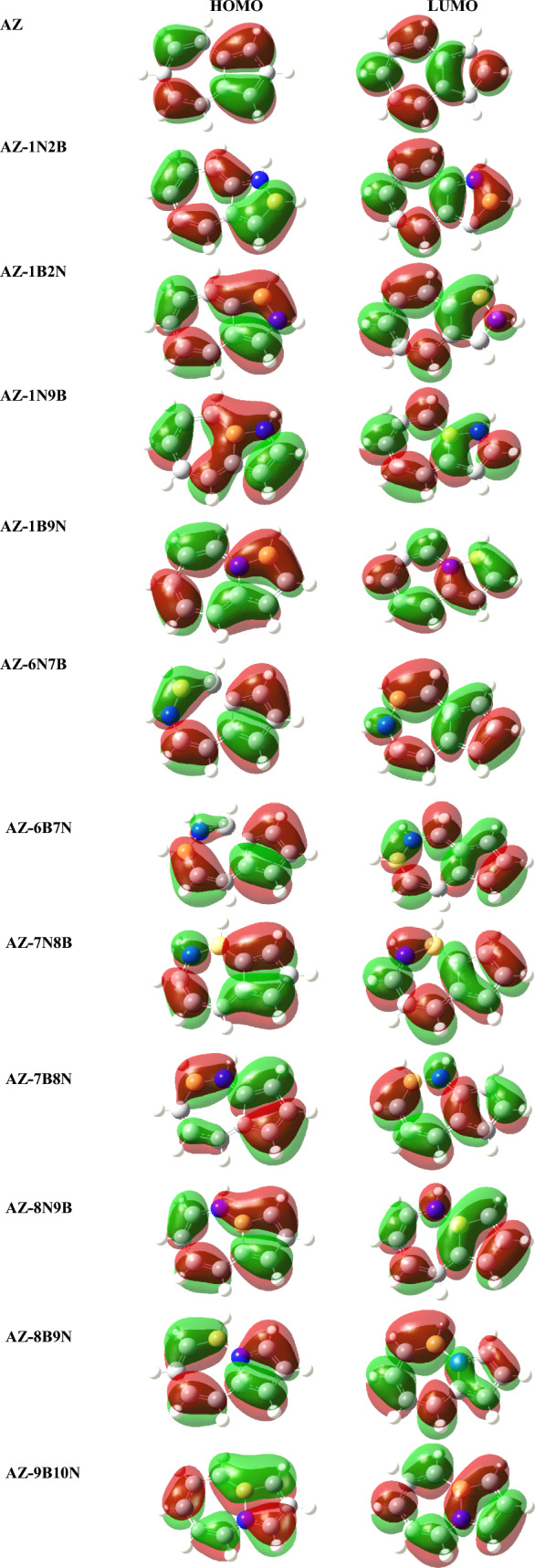
Plots of HOMOs and LUMOs distribution of BN-doped azulene using Gaussview package (version 5)^41^.

Frontier molecular orbital (FMO) energies provide useful information on electronic, optical, and electrical properties, reactivity, and kinetic stability. Figure 3 shows the HOMO–LUMO energy gap at the G3MP2 level. The corresponding plots of these orbitals are also shown in Fig. 4. The trend with respect to the HOMO energy is as follows: AZ-1B2N (− 4.99 eV) > AZ-8N9B (− 5.18 eV) > AZ-9B10N (− 5.29 eV) > AZ-6N7B (− 5.34 eV) > AZ-1B9N (− 5.38 eV) > AZ-7B8N (− 5.48 eV) > AZ-1N2B (− 5.59 eV) > AZ-6B7N (− 5.60 eV) > AZ-8B9N (− 5.69 eV) > AZ-1N9B (− 5.72 eV) > AZ-7N8B (− 5.73 eV) and the LUMO energy level ranges from − 1.84 to − 2.61 eV.

As can be noticed from Fig. 4, HOMO electron density distributions vary among the BN-substituted azulene isomers. The HOMO–LUMO energy gap is another useful parameter characteristic of the electronic and optical properties of molecules^46,47^ and provides qualitative information about the kinetic stability of the molecules^48^. Generally, a small energy gap results in a maximum absorption wavelength^49^. As shown in Fig. 3, BN substitution in azulene induces a change in the band gap. The position of BN in azulene plays a major and effective role in determining the energy gap of the molecule. Nitrogen substitution at positions 2, 6, and 8 and boron substitution at positions 1 and 7 make the molecule thermodynamically less stable and decrease the energy gap. Indeed, a large band gap in a material or molecule is often an indicator of its stability. The largest band gap calculated for the Az-1N9B isomer indicates that the molecule is very stable.

The molecular variations before and after HOMO and LUMO energy levels were displayed using DOS diagrams at the G3MP2 level for all the studied isomers (Fig. S1). The DOS provides information about the available states for the localization of charges in the energy range of − 15 to 5 eV. Figure S1 clearly shows the larger energy gap of the most stable B–N azulene-based isomers than the azulene. The less stable isomers have a smaller energy gap than azulene. Thus, azulene's B–N substitution has a significant impact on the stability.

Ionization energy (IE) and electron affinity (EA) are crucial parameters as they are used to calculate electronegativity, softness, hardness, and electrophilicity indices^38,39^. The ionization energy and electron affinity of a molecule refer to its ability to lose or accept an electron, and hence are good measures of the stability of the investigated systems toward oxidation and reduction, respectively. According to Koopmans theorem^50^, the ionization energy and electron affinity are calculated as the negative values of the HOMO and LUMO energies, respectively. In the case of DFT methods, the ionization energy calculated from orbital energies is of low accuracy, with an error of more than 2 eV depending on the used exchange–correlation functionals^51,52^. The LUMO energies for electron affinities are even worse^53^. Table 2 lists ionization energies and electron affinities from HOMO and LUMO energies for 11 isomers of BN-doped azulene, as well as the energies from the adiabatic approach which takes into account the geometrical rearrangement of the molecular cation and anion after electron loss and acceptance, respectively. The vertical ionization energy (VIE) can be obtained by the energy difference between a neutral molecule and its cation with the molecular cation at the same geometry as the neutral molecule. Electron affinity is similar to ionization energy, but adiabatic electron affinity (AEA) and vertical electron affinity (VEA) can be obtained from molecular anions. The higher value of AIE indicates the higher stability of the molecule against oxidation. The electron affinities calculated from Koopmans theory for azulene and its isosteric isomers were all negative. However, positive electron affinities have been reported for azulene in experiments. Therefore, the AEAs for azulene and all BN-doped structures giving positive values were considered. The agreement between the experimental and calculated adiabatic electron affinity for the parent azulene is very good. It is obvious that the most stable isostere, AZ-1N9B, has the greatest AIE (7.85 eV), which decreases to 7.25 eV in the least stable isostere (AZ-9B10N). On the other hand, the lowest AEA value of AZ-1N9B is 0.50 eV, which increases to 0.86 eV in the AZ-9B10N. That is proven by the hardness, the least reactivity, and the stability of AZ-1N9B isostere.

**Table 2:** Ionization energies, electron affinities (Koopmans IE, EA), adiabatic ionization energies, and electron affinities (AIE, AEA) calculated using G3MP2 method.

Systems	IE (eV)	AIE (eV)	Exp. IE (eV)	EA (eV)	AEA (eV)	Exp. EA (eV)
AZ	7.12	7.66	7.38 ± 0.03^54^, 7.32 ± 0.05^55^	− 1.00	0.68	0.79 ± 0.008^56^, 0.69 ± 0.10^57^, 0.80 ± 0.10^58^
AZ-1N2B	7.13	–	–	− 0.96	0.61	–
AZ-1B2N	6.58	7.15	–	− 1.01	0.79	–
AZ-1N9B	7.36	7.85	–	− 1.02	0.50	–
AZ-1B9N	7.10	7.56		− 0.92	0.93	–
AZ-6N7B	7.10	7.39	–	− 0.88	0.89	–
AZ-6B7N	7.36	7.60	–	− 0.87	0.46	–
AZ-7N8B	7.56	7.79	–	− 0.87	0.79	–
AZ-7B8N	7.43	7.44	–	− 0.57	1.18	–
AZ-8N9B	6.85	7.35	–	− 0.95	0.59	–
AZ-8B9N	7.35	7.77	–	− 1.01	0.63	–
AZ-9B10N	6.51	7.25	–	− 0.57	0.86	–

**Figure 5:**
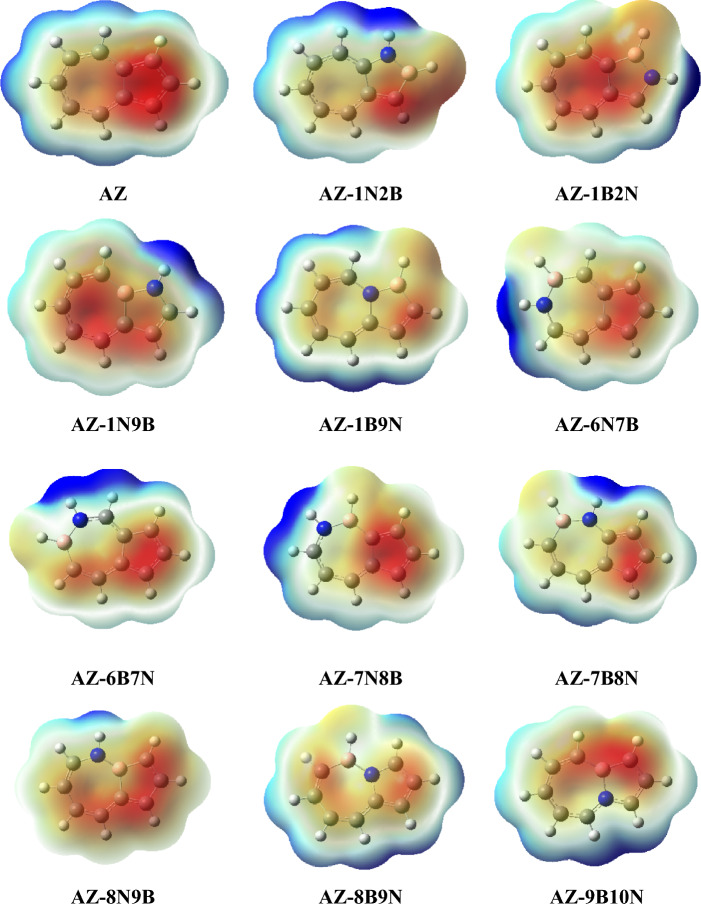
Molecular ESP surfaces of azulene and its BN-doped isosteres at B3LYP/6–31 + G(d,p) level using Gaussview package (version 5)^41^.

**Figure 6:**
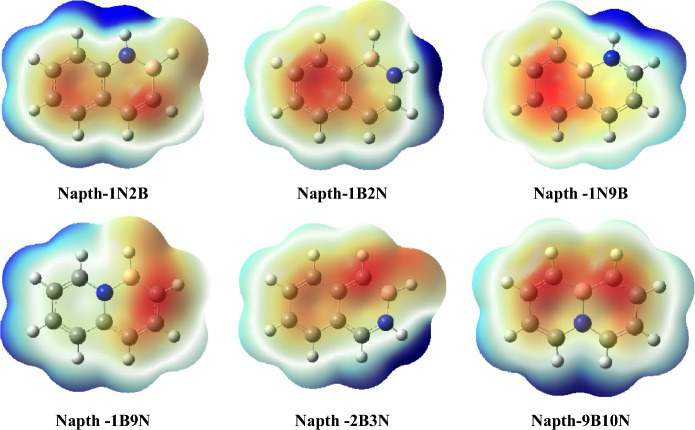
Molecular ESP surfaces of naphthalene and its BN-doped isosteres at B3LYP/6–31 + G(d,p) level using Gaussview package (version 5)^41^.

The reactivity of a given molecule can be monitored by plotting the electrostatic potential (ESP). Figures 5 and 6 display the ESP surfaces of azulene, naphthalene, and their BN-doped isosteres at B3LYP/6–31 + G(d,p). ESP maps depict the charged regions in the molecule with different colors, which reflect values of the electrostatic potential. The red color in ESP maps represents the most negative electrostatic potential, while the blue indicates the most positive electrostatic potential regions. The azulene compound has a negative charge on the five-membered ring and a positive charge on the seven-membered ring (see Fig. 5). ESP of the most stable structure, AZ-1N9B, shows the localization of a significant negative charge on the seven-membered ring and the appearance of the blue color around the H atom bonded to the nitrogen. The higher electron-donating ability of the heptagonal ring, as opposed to the electron-accepting ability of 1N-H, might provide evidence of the stability of the AZ-1N9B structure. In AZ-1B9N, AZ-8B9N, and AZ-9B10N, one fused carbon is substituted with a nitrogen atom, resulting in the dispersion of the positive charge around the structure and consequently the negative charge as well. This charge dispersion explains the lower stability of AZ-1B9N, AZ-8B9N, and AZ-9B10N.

Before the absorption wavelengths calculation for the investigated 11 isomers BN-doped azulene in the gas phase and acetone, we have carried out benchmark calculations to assess the impact of the basis set, functionals, and dispersion corrections. We have examined B3LYP^35^, CAM-B3LYP^40^, wB97XD^59^, HSEH1PBE (HSE06)^60^, PBE0DH^60^, MN15^61^, MN12-SX^62^, and B97D^63^ functionals. These are the recently developed and most accurate functionals reported to date. The dispersion corrections GD3 of Grimme with the original D3 damping parameter^64^, and GD3BJ with the Becke–Johnson damping D3 parameter^65^. We also tested several basis sets; 6−311G(d,p), 6–311 + G(d,p), cc-pVTZ, cc-pVQZ, and Def2TZVPP. The results of these benchmark calculations are shown in Fig. S2, while the optimized structures are collected in Table S3 in SI file. We did not observe any impact of using different basis sets, functionals, or dispersion corrections on the optimized geometries of the most stable isomer AZ-1N9B. Therefore, we limit the optimization level to B3LYP/6–31 + G(d,p), and the *λ*_max_ values were calculated at TD-B3LYP/6–31 + G(d,p) and summarized in Tables 3 and 4, respectively. According to Tawada et al.^66^, long-range corrected DFT should be used for computing the charge transfer excited states in TD-DFT. Therefore, Table S4 lists the *λ*_max_ values, and Fig. S3 depicts the absorption spectra of the different isomers calculated using TD-CAM-B3LYP/6–31 + G(d,p) level. Table S5 reported the calculated and previously studied transition wavelengths for S_0_ → S_1_ and S_0_ → S_2_ electronic transitions for azulene. Our calculated S_0_ → S_1_ and S_0_ → S_2_ electronic transitions for azulene at CAM-B3LYP are in good agreement with those previously calculated at TD-PBE0/6–31G(d)^67^. For S_0_ → S_1_ and S_0_ → S_2_, the computed wavelength in acetone with TD-B3LYP are longer by 9 and 16 nm with respect to the TD-CAM-B3LYP estimates, however, TD-B3LYP shows great accordance with the S_0_ → S_2_ experimental result^68^ (within 2 nm eV). In the case of S_0_ → S_1_, both TD-B3LYP and TD-CAM-B3LYP have close wavelengths being both underestimated by up to 60 nm with respect to the experimental values. Therefore, the TD-B3LYP method will be used through the discussion of the UV absorption spectra of the studied isomers.

**Table 3:** Computed absorption spectra (*λ*_max_, nm/eV) of azulene and BN-doped azulene at TD-DFT(PCM)/B3LYP/6–31 + G(d,p) in acetone.

Compound	Absorption energy		*F*	Transitions
	(nm)	(eV)		
AZ	264	4.70	1.2649	H-1 → L (55%), H → L + 1 (44%)
AZ-1N2B	249	4.99	0.7499	H-1 → L (12%), H-1 → L + 1 (70%)
AZ-1B2N	250	4.96	0.3154	H-2 → L (10%), H-1 → L (38%), H → L + 5 (39%)
AZ-1N9B	213	5.82	0.5386	H-2 → L (73%), H-1 → L + 1 (15%)
AZ-1B9N	278	4.46	0.8577	H-1 → L (52%), H → L + 1 (39%)
AZ-6N7B	269	4.61	0.6006	H-2 → L (29%), H-1 → L (34%), H → L + 1 (30%)
AZ-6B7N	263	4.72	1.0129	H-1 → L (66%), H → L + 1 (20%)
AZ-7N8B	252	4.93	0.6146	H-1 → L + 1 (80%)
AZ-7B8N	284	4.37	0.8134	H-1 → L (31%), H → L + 1 (65%)
AZ-8N9B	250	4.96	0.4542	H-1 → L + 1 (89%)
AZ-8B9N	259	4.79	0.5616	H-2 → L (10%), H-1 → L (53%), H → L + 1 (33%)
AZ-9B10N	287	4.32	0.5006	H-1 → L (58%), H → L + 1 (31%)

**Table 4:** Absorption spectra (*λ*_max_, nm/eV) of azulene and BN-doped azulene calculated at TD-DFT/B3LYP/6–31 + G(d,p) in the gas phase.

Compound	Absorption energy		*F*	Transitions
	(nm)	(eV)		
AZ	252	4.92	1.0782	H-1 → L (53%), H → L + 1 (45%)
AZ-1N2B	242	5.13	0.7340	H-1 → L (22%), H-1 → L + 1 (53%), H → L + 1 (11%)
AZ-1B2N	235	5.27	0.3766	H-2 → L (32%), H-1 → L (22%), H-1 → L + 1 (32%)
AZ-1N9B	247	5.02	0.3534	H-2 → L (22%), H-1 → L + 1 (64%)
AZ-1B9N	268	4.63	0.6860	H-1 → L (47%), H → L + 1 (41%)
AZ-6N7B	241	5.14	0.5392	H-2 → L (41%), H-1 → L (26%), H → L + 1 (17%)
AZ-6B7N	253	4.89	0.8507	H-1 → L (62%), H → L + 1 (23%)
AZ-7N8B	248	5.01	0.5734	H-1 → L + 1 (65%), H → L + 1 (18%)
AZ-7B8N	275	4.51	0.6441	H-1 → L (33%), H → L + 1 (59%)
AZ-8N9B	246	5.04	0.3888	H-1 → L + 1 (86%)
AZ-8B9N	254	4.88	0.4067	H-2 → L (16%), H-1 → L (44%), H → L + 1 (34%)
AZ-9B10N	281	4.41	0.3568	H-1 → L (53%), H → L + 1 (30%)

**Figure 7:**
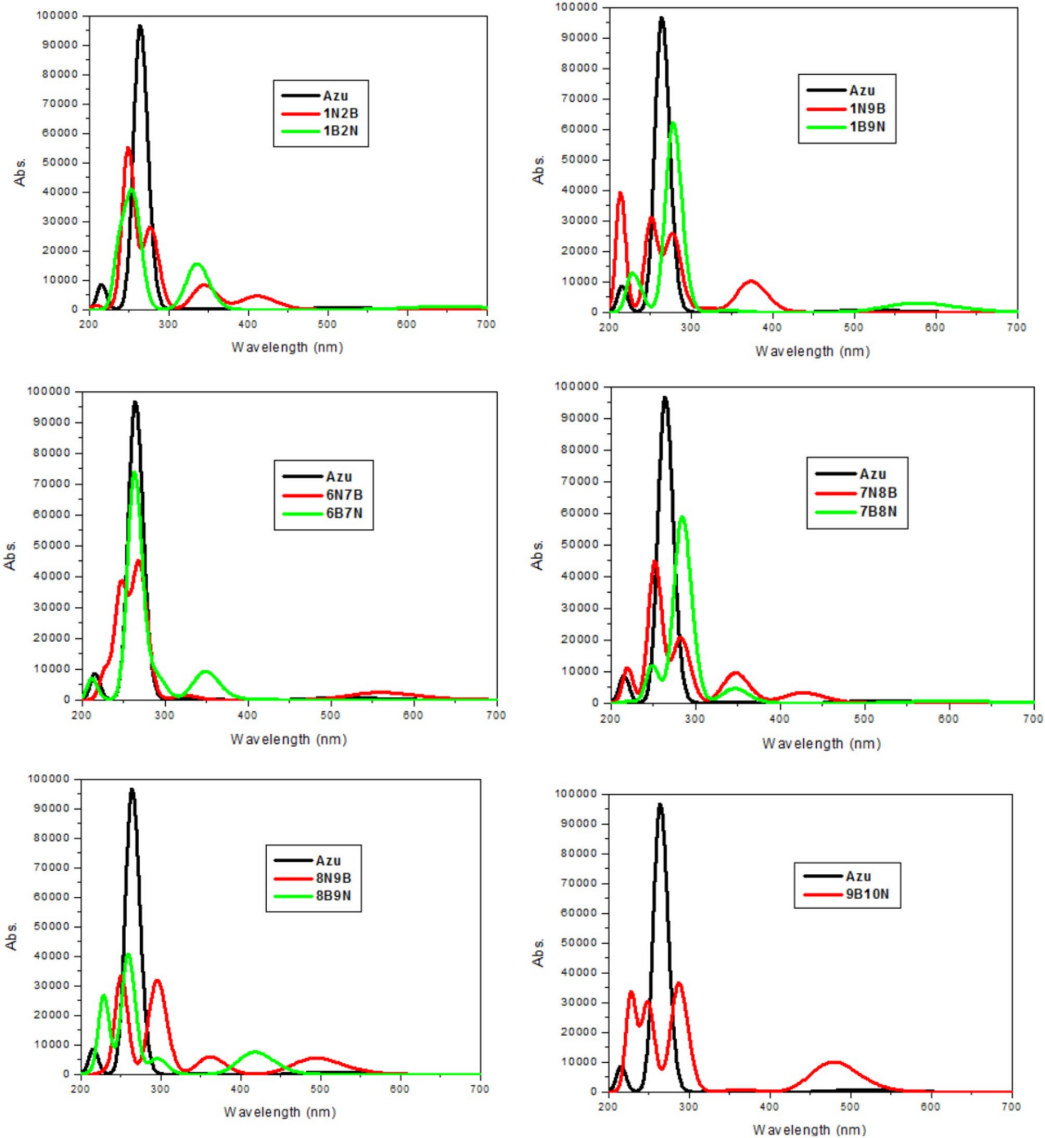
UV–Vis spectra for azulene and BN-doped azulene at TD-DFT/6–31 + G(d,p)(PCM)//B3LYP/6−31G(d,p) in acetone.

Results of the absorption wavelengths reported in Tables 3 and 4, reveal a red shift of about 4–15 nm in all calculated wavelengths except for the AZ-1N9B isomer, in which we observed a blue shift. This is expected as the molecule exhibits the highest stability and the highest band gap. The UV–Vis absorption spectra are shown in Fig. 7 for S0–S1 transition calculated at TD-B3LYP and TD-CAM-B3LYP levels, respectively. As shown in Fig. 7, the electronic transition can be referred to as that of π → π* and n → π* transitions. The most intense absorption peaks of azulene are in a lower wavelength region, mostly below 300 nm. All BN-doped azulene isomers show an extension of the absorption regions above 300 nm, to be in the UV and visible regions of the electromagnetic spectrum. The AZ-1N9B isomer gives the shortest *λ*_max_ but the highest extension at the visible region by having a peak centered at 578 nm. AZ-8N9B, AZ-9B10N, and AZ-6N7B exhibit intense peaks centered at 495, 479, and 560 nm, respectively.

## Conclusion

The thermodynamic stabilities and opt-electronic properties of new series of BN-doped azulene molecules are investigated. To do so, we have calculated formation enthalpies of 11 BN-substituted azulenes at three computational levels. Based on the available experimental data, the formation enthalpy obtained from G3MP2 and CBS-QB3 is more accurate than those estimated from the CBS-APNO model chemistry. Our calculations reveal that the most stable is AZ-1N9B isomer with the largest HOMO–LUMO gap and lowest formation enthalpy among all other isomers. Ionization energies and electron affinities were estimated by the vertical approach in the G3MP2 model. AZ-7N8B, AZ-7B8N, AZ-6B7N, and AZ-1N9B isomers were found to give higher ionization energies. Moreover, the electronic absorption spectra of these isomers have been determined in the gas phase and in acetone. The absorption wavelength in acetone displays a red shift compared with the gas phase except for the AZ-1N9B isomer which exhibits a blue shift. The BN vicinal doping of azulene populates the visible region with absorption peaks compared to the parent azulene, which increases the light-harvesting capacity of the doped molecules.

## Supplementary Information

Supplementary Information.

## Data Availability

All data generated through this study are included in this manuscript and the Supporting Information file.
