# Supplementary material for: Ab initio calculations on structure and stability of BN/CC isosterism in azulene
Source: Sci Rep. 2023 Jun 24;13:10260. doi: 10.1038/s41598-023-37047-7 (PMC10290675; doi:10.1038/s41598-023-37047-7)
Supplement: Supplementary file 1 — Supplementary Information. [file 41598_2023_37047_MOESM1_ESM.docx]

**Ab Initio Calculations of BN/CC isosterism in Azulene: Structure and Stability**

Mohamed A. Abdel-Rahman,^a*^ Kamal A. Soliman,^b*^ Safwat Abdel-Azeim,^c^ Ahmed M. El-Nahas,^d^ Tetsuya Taketsugu,^e,f^ Takahito Nakajima,^g^ Asmaa B. El-Meligy^d^*

^a^Chemistry Department, Faculty of Science, Suez University, Suez, 43518, Egypt

^b^Chemistry Department, Faculty of Science, Benha University, Benha 13518, Egypt

^c^ Center for Integrative Petroleum Research (CIPR), College of Petroleum Engineering and Geosciences, King Fahd University of Petroleum and Minerals (KFUPM), Dhahran 31261, Saudi Arabia

^d^ Chemistry Department, Faculty of Science, Menoufia University, Shebin El-Kom, 32512, Egypt

^e^Department of Chemistry, Faculty of Science, Hokkaido University, Sapporo 060-0810, Japan

^f^Institute for Chemical Reaction Design and Discovery (WPI-ICReDD), Hokkaido University, Sapporo 060-0810, Japan

^g^RIKEN, Center for Computational Science, 7-1-26 Minatojima-minami, Chuo Kobe 650-0047, Japan

**Corresponding author:**

Mohamed.Abdel-Rahman@sci.suezuni.edu.eg (Mohamed A. Abdel-Rahman)

kamal.soliman@fsc.bu.edu.eg (Kamal A. Soliman)

[asmaaphys@yahoo.com](mailto:asmaaphys@yahoo.com) (Asmaa B. El-Meligy)

Table S1: The Cartesian coordinates of azulene and 11 isomers BN-doped azulene at B3LYP/6-311G(d,p) (optimization level of CBS-QB3 method)

| 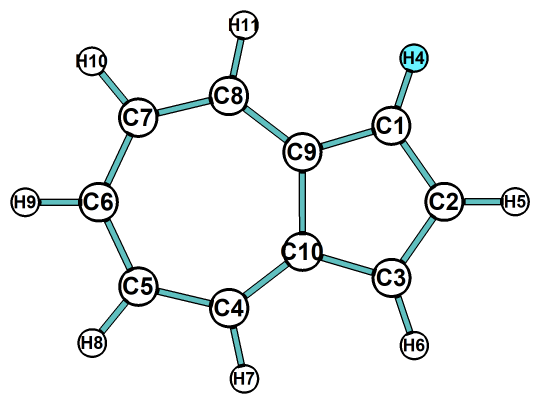 | AZ | \| C \| 1.913224 \| 1.267476 \| -9.5E-05 \| \| --- \| --- \| --- \| --- \| \| C \| 0.552611 \| 1.59605 \| -0.0001 \| \| C \| -0.55472 \| 0.750175 \| -6.7E-05 \| \| C \| -0.55469 \| -0.75005 \| 0.000045 \| \| H \| 3.595395 \| 0.00016 \| 0.000147 \| \| H \| 2.601116 \| 2.109515 \| -6.9E-05 \| \| H \| 0.324416 \| 2.661473 \| -7.4E-05 \| \| H \| 2.601042 \| -2.1097 \| 0.000129 \| \| H \| 0.324174 \| -2.66134 \| 0.000144 \| \| C \| -1.90437 \| 1.150935 \| 0.000147 \| \| H \| -2.251 \| 2.177135 \| 0.000259 \| \| C \| -2.71185 \| -4.9E-05 \| -7.1E-05 \| \| H \| -3.79623 \| -0.00012 \| -0.00011 \| \| C \| -1.90426 \| -1.15099 \| 0.000001 \| \| H \| -2.25083 \| -2.17722 \| -1.4E-05 \| \| C \| 2.506825 \| -6E-06 \| -7.8E-05 \| \| C \| 0.552623 \| -1.59596 \| 0.000095 \| \| C \| 1.913259 \| -1.26757 \| 0.000055 \| |
| --- | --- | --- | --- | --- | --- | --- | --- | --- | --- | --- | --- | --- | --- | --- | --- | --- | --- | --- | --- | --- | --- | --- | --- | --- | --- | --- | --- | --- | --- | --- | --- | --- | --- | --- | --- | --- | --- | --- | --- | --- | --- | --- | --- | --- | --- | --- | --- | --- | --- | --- | --- | --- | --- | --- | --- | --- | --- | --- | --- | --- | --- | --- | --- | --- | --- | --- | --- | --- | --- | --- | --- | --- | --- | --- |
| 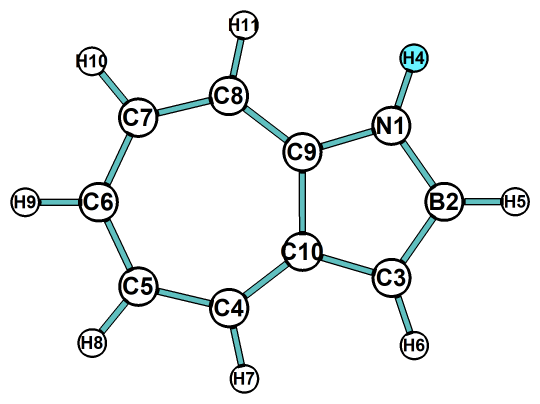 | AZ-1N2B | \| C \| 1.921042 \| 1.240468 \| -2.5E-05 \| \| --- \| --- \| --- \| --- \| \| C \| 0.592826 \| 1.602013 \| -3.9E-05 \| \| C \| -0.58035 \| 0.790896 \| -2.2E-05 \| \| C \| -0.57209 \| -0.69581 \| 0.00002 \| \| H \| 3.593123 \| -0.07643 \| -2.1E-05 \| \| H \| 2.630841 \| 2.06492 \| -2.6E-05 \| \| H \| 0.391427 \| 2.671955 \| -5.2E-05 \| \| H \| 2.527448 \| -2.15295 \| 0.000046 \| \| H \| 0.228977 \| -2.63047 \| 0.000048 \| \| C \| -1.89177 \| 1.243509 \| -1.4E-05 \| \| H \| -2.12742 \| 2.302306 \| -2.4E-05 \| \| H \| -3.98239 \| -0.11691 \| -9E-06 \| \| H \| -2.10326 \| -2.08979 \| -9E-06 \| \| C \| 2.505913 \| -0.0554 \| 0.000021 \| \| C \| 0.488032 \| -1.57249 \| 0.00004 \| \| C \| 1.876785 \| -1.28129 \| 0.00004 \| \| N \| -1.88555 \| -1.10363 \| -7E-06 \| \| B \| -2.80045 \| 0.024275 \| -4E-06 \| |
| 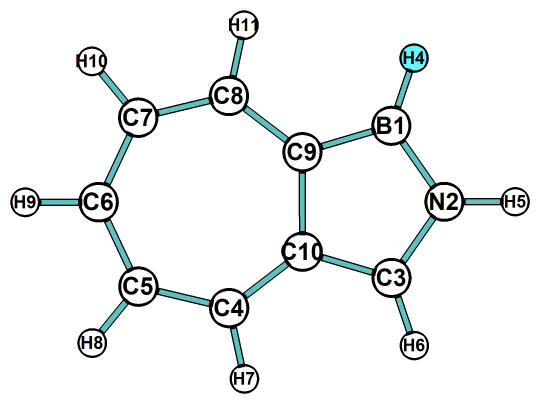 | AZ-1B2N | \| C \| 1.912293 \| 1.284813 \| -8.3E-05 \| \| --- \| --- \| --- \| --- \| \| C \| 0.574577 \| 1.589557 \| -0.00012 \| \| C \| -0.54212 \| 0.703948 \| -5.2E-05 \| \| C \| -0.52001 \| -0.79343 \| 0.000052 \| \| H \| 3.62266 \| 0.017475 \| -5.4E-05 \| \| H \| 2.598986 \| 2.127454 \| -0.00007 \| \| H \| 0.325452 \| 2.651011 \| -0.00011 \| \| H \| 2.6649 \| -2.09818 \| 0.000086 \| \| H \| 0.376515 \| -2.67813 \| 0.000088 \| \| C \| -1.85528 \| 1.106869 \| 0.000103 \| \| H \| -2.23994 \| 2.119163 \| 0.000155 \| \| H \| -3.71745 \| 0.151696 \| -0.00012 \| \| H \| -2.51327 \| -2.3047 \| -8.1E-05 \| \| C \| 2.534049 \| -0.00752 \| 0.000044 \| \| C \| 0.578539 \| -1.60709 \| 0.000092 \| \| C \| 1.972989 \| -1.25908 \| 0.000087 \| \| B \| -2.00397 \| -1.22946 \| -3.8E-05 \| \| N \| -2.71832 \| 0.007586 \| -6.4E-05 \| |
| 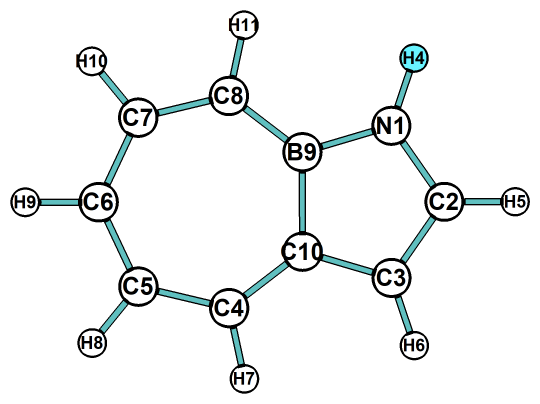 | AZ-1N9B | \| C \| 1.902515 \| 1.294336 \| 0.000001 \| \| --- \| --- \| --- \| --- \| \| C \| 0.534555 \| 1.647875 \| 0.000004 \| \| C \| -0.5472 \| 0.781497 \| 0.000008 \| \| H \| 3.610745 \| 0.092383 \| 0.000003 \| \| H \| 2.589046 \| 2.139569 \| -3.3E-05 \| \| H \| 0.331997 \| 2.720389 \| -3.5E-05 \| \| H \| 2.763891 \| -2.04396 \| 0.000025 \| \| H \| 0.541501 \| -2.77479 \| 0.000032 \| \| C \| -1.93653 \| 1.14629 \| -8.7E-05 \| \| H \| -2.34292 \| 2.151231 \| -0.00015 \| \| H \| -3.78036 \| -0.06633 \| 0.000003 \| \| H \| -2.35625 \| -2.05745 \| 0.000084 \| \| C \| 2.523311 \| 0.049231 \| 0.000016 \| \| C \| 1.996407 \| -1.26865 \| 0.000011 \| \| C \| -2.69788 \| 0.000027 \| -5E-06 \| \| B \| -0.51788 \| -0.77903 \| 0.000003 \| \| N \| -1.9256 \| -1.14573 \| 0.000042 \| \| C \| 0.676634 \| -1.69158 \| 0.000011 \| |
| 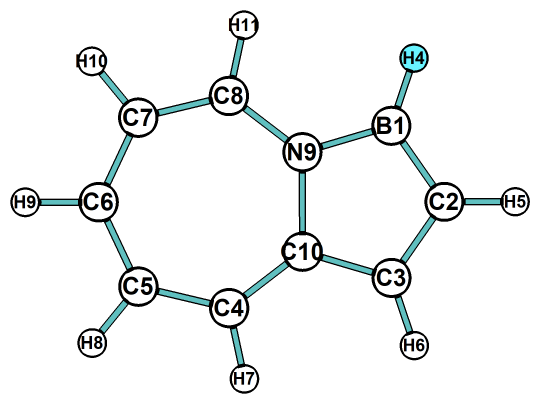 | AZ-1B9N | \| C \| -1.91183 \| 1.28225 \| 0.000001 \| \| --- \| --- \| --- \| --- \| \| C \| -0.49707 \| 1.547656 \| -0.00014 \| \| C \| 0.564476 \| 0.695887 \| -0.00016 \| \| H \| -3.60238 \| 0.050606 \| -0.00043 \| \| H \| -2.55354 \| 2.158848 \| 0.000232 \| \| H \| -0.21676 \| 2.598755 \| -8E-06 \| \| H \| -2.56405 \| -2.09091 \| 0.000016 \| \| H \| -0.3082 \| -2.61509 \| 0.000117 \| \| C \| 1.940941 \| 1.104775 \| 0.000144 \| \| H \| 2.212582 \| 2.156053 \| 0.000268 \| \| H \| 3.870913 \| 0.134011 \| -9.5E-05 \| \| H \| 2.175941 \| -2.39641 \| -0.0002 \| \| C \| -2.5146 \| 0.059716 \| 0.000025 \| \| C \| -1.8907 \| -1.2393 \| 0.000108 \| \| C \| 2.791583 \| 0.027219 \| -4.4E-05 \| \| C \| -0.5683 \| -1.56016 \| 0.000135 \| \| N \| 0.557779 \| -0.76189 \| 0.000027 \| \| B \| 1.91881 \| -1.23417 \| -0.0001 \| |
| 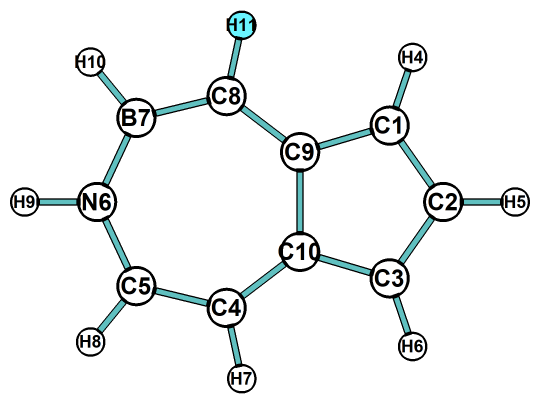 | AZ-6N7B | \| C \| -1.95821 \| -1.19672 \| -2.8E-05 \| \| --- \| --- \| --- \| --- \| \| C \| -0.64031 \| -1.54921 \| -2.4E-05 \| \| C \| 0.532203 \| -0.73813 \| -1.1E-05 \| \| C \| -0.42001 \| 1.658325 \| 0.000031 \| \| C \| 0.615892 \| 0.76786 \| 0.000011 \| \| H \| -3.51727 \| 0.054216 \| 0.000041 \| \| H \| -2.69568 \| -1.99363 \| -1.8E-05 \| \| H \| -0.45916 \| -2.62187 \| -3.5E-05 \| \| H \| -2.71808 \| 2.27519 \| 0.000049 \| \| H \| -0.11858 \| 2.707614 \| 0.000058 \| \| C \| 1.830246 \| -1.20439 \| -0.00004 \| \| H \| 2.125583 \| -2.24706 \| -7.1E-05 \| \| C \| 2.736659 \| -0.08162 \| 0.000011 \| \| H \| 3.817955 \| -0.16593 \| 0.000015 \| \| C \| 2.024368 \| 1.092527 \| 0.000016 \| \| H \| 2.420401 \| 2.100066 \| 0.000033 \| \| B \| -1.92917 \| 1.376353 \| 0.000026 \| \| N \| -2.50491 \| 0.073967 \| 0.000002 \| |
| 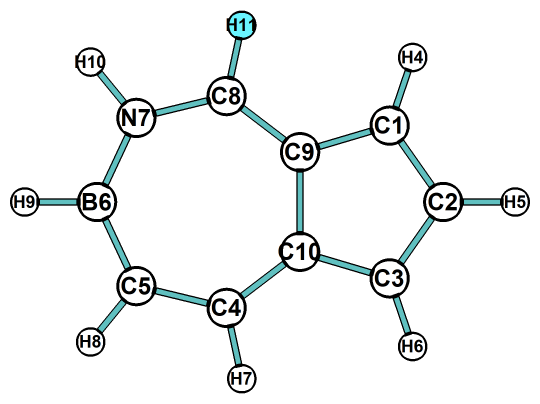 | AZ-6B7N | \| C \| -1.90563 \| 1.363583 \| -0.00014 \| \| --- \| --- \| --- \| --- \| \| C \| -0.56232 \| 1.662974 \| -0.00011 \| \| C \| 0.544612 \| 0.769446 \| 0.000004 \| \| C \| -0.54701 \| -1.57157 \| 0.000151 \| \| C \| 0.520007 \| -0.71224 \| 0.000106 \| \| H \| -3.76552 \| -0.10866 \| -6.8E-05 \| \| H \| -2.56779 \| 2.22894 \| -0.00021 \| \| H \| -0.27788 \| 2.716925 \| -4.7E-05 \| \| H \| -2.46751 \| -2.08883 \| 0.000077 \| \| H \| -0.32879 \| -2.63794 \| 0.000097 \| \| C \| 1.88532 \| 1.139553 \| 0.000348 \| \| H \| 2.24771 \| 2.161171 \| 0.000612 \| \| C \| 2.702887 \| -0.033 \| -0.0002 \| \| H \| 3.78645 \| -0.03807 \| -0.00028 \| \| C \| 1.890066 \| -1.15045 \| -0.00019 \| \| H \| 2.20919 \| -2.18587 \| -0.00033 \| \| N \| -1.87583 \| -1.26722 \| 0.000078 \| \| B \| -2.57453 \| 0.002616 \| -4.1E-05 \| |
| 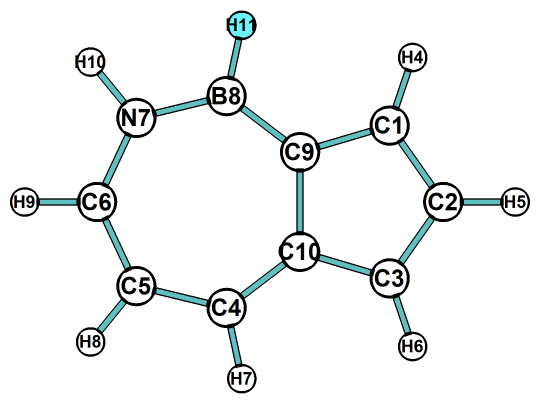 | AZ-7N8B | \| C \| 1.843627 \| 1.304788 \| 0.000079 \| \| --- \| --- \| --- \| --- \| \| C \| 0.444484 \| 1.578779 \| 0.000261 \| \| C \| -0.61725 \| 0.704538 \| 0.000225 \| \| C \| -0.57164 \| -0.78089 \| -3.8E-05 \| \| H \| 3.594612 \| 0.144867 \| 0.000504 \| \| H \| 2.494231 \| 2.17453 \| -0.00013 \| \| H \| 0.190015 \| 2.638425 \| 0.000124 \| \| H \| 2.726769 \| -1.87375 \| -0.00019 \| \| H \| 0.561136 \| -2.86384 \| -0.00022 \| \| C \| -2.0024 \| 1.092905 \| -0.00038 \| \| H \| -2.36131 \| 2.11551 \| -0.00069 \| \| C \| -2.76875 \| -0.05484 \| 0.000085 \| \| H \| -3.85216 \| -0.09605 \| 0.000184 \| \| C \| -1.89582 \| -1.19666 \| 0.000229 \| \| H \| -2.22439 \| -2.23001 \| 0.00046 \| \| C \| 2.507997 \| 0.103861 \| -7.4E-05 \| \| B \| 0.643666 \| -1.67063 \| -0.00021 \| \| N \| 2.001616 \| -1.16734 \| -0.00019 \| |
| 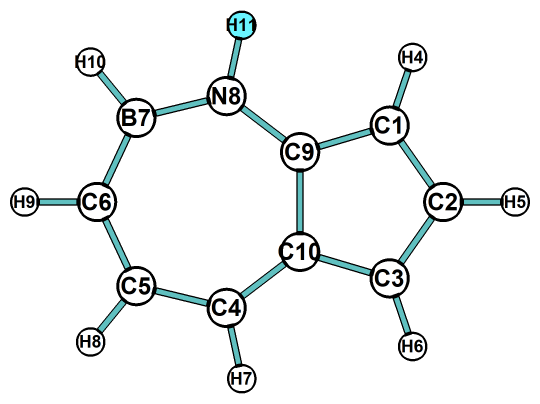 | AZ-7B8N | \| C \| -1.97751 \| 1.260856 \| 0.000043 \| \| --- \| --- \| --- \| --- \| \| C \| -0.57657 \| 1.574065 \| 0.000097 \| \| C \| 0.513496 \| 0.744294 \| 0.000077 \| \| C \| 0.567861 \| -0.75263 \| -3.5E-05 \| \| H \| -3.66454 \| 0.052953 \| 0.00017 \| \| H \| -2.62482 \| 2.137797 \| -8.6E-05 \| \| H \| -0.34382 \| 2.638394 \| 0.000052 \| \| H \| -2.60058 \| -2.35071 \| -6.8E-05 \| \| H \| -0.24472 \| -2.56849 \| -0.00012 \| \| C \| 1.886487 \| 1.18419 \| -1.6E-05 \| \| H \| 2.198101 \| 2.220481 \| -3.9E-05 \| \| C \| 2.69422 \| 0.071438 \| 0.000032 \| \| H \| 3.778369 \| 0.078671 \| 0.000055 \| \| C \| 1.88973 \| -1.127 \| 0.000009 \| \| H \| 2.265874 \| -2.14297 \| 0.000015 \| \| B \| -1.92646 \| -1.36309 \| -9.2E-05 \| \| N \| -0.52443 \| -1.59285 \| -9.5E-05 \| \| C \| -2.57448 \| 0.027995 \| -1.8E-05 \| |
| 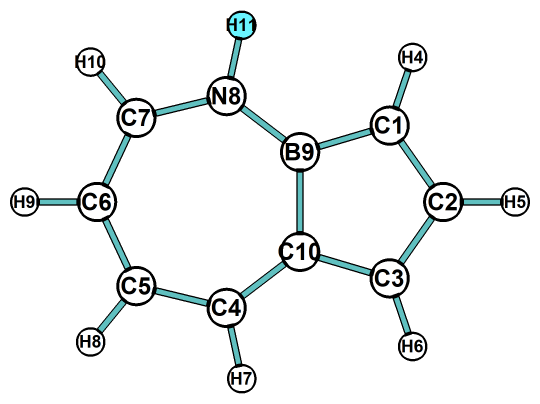 | AZ-8N9B | \| C \| -1.89514 \| 1.292292 \| 0.000013 \| \| --- \| --- \| --- \| --- \| \| C \| -0.57121 \| 1.681285 \| 0.000028 \| \| C \| 0.553684 \| 0.820068 \| 0.000019 \| \| H \| -3.56313 \| -0.02959 \| 0.000025 \| \| H \| -2.62641 \| 2.097977 \| 0.000023 \| \| H \| -0.38385 \| 2.756041 \| 0.000051 \| \| H \| -2.59352 \| -2.11924 \| -5.3E-05 \| \| H \| -0.42234 \| -2.61128 \| -5.4E-05 \| \| C \| 1.882085 \| 1.177111 \| 0.000034 \| \| H \| 2.268037 \| 2.193527 \| 0.000055 \| \| H \| 3.828542 \| 0.081124 \| 0.000025 \| \| H \| 2.538731 \| -2.1586 \| -2.4E-05 \| \| C \| -2.47604 \| -0.01684 \| -2.1E-05 \| \| C \| -1.91916 \| -1.26779 \| -0.00004 \| \| C \| 2.049175 \| -1.19018 \| -1.1E-05 \| \| C \| 2.743182 \| -0.00448 \| 0.000017 \| \| B \| 0.564454 \| -0.77742 \| -8E-06 \| \| N \| -0.58111 \| -1.61024 \| -3.6E-05 \| |
| 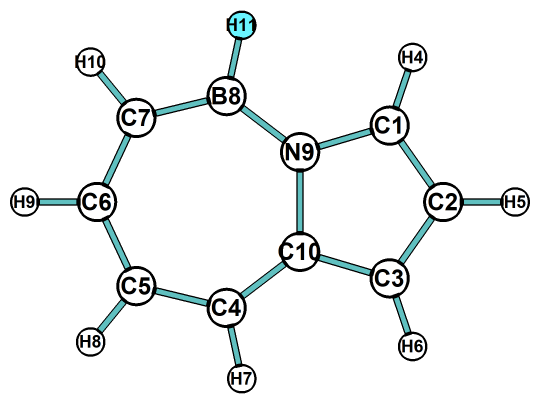 | AZ-8B9N | \| C \| 1.929598 \| 1.251682 \| -3.1E-05 \| \| --- \| --- \| --- \| --- \| \| C \| 0.589403 \| 1.541607 \| -5.4E-05 \| \| C \| -0.56065 \| 0.706408 \| -3.2E-05 \| \| H \| 3.659162 \| 0.020493 \| 0.000015 \| \| H \| 2.597727 \| 2.109247 \| -5.1E-05 \| \| H \| 0.331043 \| 2.598844 \| -8.2E-05 \| \| H \| 2.70407 \| -2.10572 \| 0.000078 \| \| H \| 0.18819 \| -2.80151 \| 0.000063 \| \| C \| -1.88492 \| 1.14377 \| -2.3E-05 \| \| H \| -2.19242 \| 2.180886 \| -3.3E-05 \| \| H \| -3.80322 \| -0.00552 \| 0.000078 \| \| H \| -2.15187 \| -2.15503 \| -0.00007 \| \| C \| 2.569606 \| -0.02957 \| 0.000032 \| \| C \| 1.996724 \| -1.2767 \| 0.000057 \| \| C \| -1.90189 \| -1.10455 \| -4.7E-05 \| \| C \| -2.72195 \| 0.006956 \| 0.000041 \| \| N \| -0.57855 \| -0.71519 \| 0.000014 \| \| B \| 0.524331 \| -1.6546 \| 0.00005 \| |
| 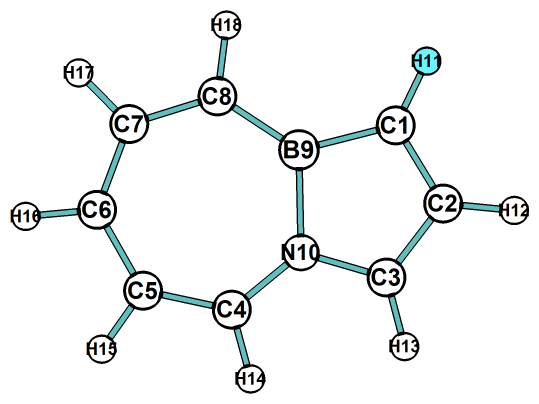 | AZ-9B10N | \| C \| -1.82142 \| -1.29075 \| -2.5E-05 \| \| --- \| --- \| --- \| --- \| \| C \| -0.47129 \| -1.57588 \| -4.5E-05 \| \| H \| -3.60735 \| -0.16822 \| -2.2E-05 \| \| H \| -2.44593 \| -2.18066 \| 0.000004 \| \| H \| -0.18293 \| -2.62561 \| -4.6E-05 \| \| H \| -2.80531 \| 2.015487 \| 0.000064 \| \| H \| -0.56231 \| 2.74263 \| 0.000071 \| \| C \| 1.87547 \| -1.08357 \| -5E-06 \| \| H \| 2.152691 \| -2.13173 \| -1.4E-05 \| \| H \| 3.815252 \| -0.06436 \| -8.6E-05 \| \| H \| 2.483094 \| 2.203674 \| 0.000048 \| \| C \| -2.5247 \| -0.06518 \| 0.000017 \| \| C \| -2.03899 \| 1.24064 \| 0.00005 \| \| C \| 2.734597 \| 0.040668 \| -5.5E-05 \| \| C \| -0.69931 \| 1.660732 \| 0.00005 \| \| B \| 0.542628 \| 0.847242 \| 0.00002 \| \| C \| 2.012278 \| 1.227435 \| 0.000023 \| \| N \| 0.577114 \| -0.70742 \| -2.5E-05 \| |

**Table S2.** Relative energies (298 K, kcal/mol) of 11 BN-doped azulene at CBS-QB3, G3MP2, and CBS-APNO methods.

| Isomer | CBS-QB3 | G3MP2 | CBS-APNO |
| --- | --- | --- | --- |
| AZ-1N9B | 0.00 | 0.00 | 0.00 |
| AZ-1N2B | 3.64 | 4.15 | 3.23 |
| AZ-8B9N | 3.74 | 4.61 | 3.66 |
| AZ-7N8B | 6.10 | 6.32 | 6.00 |
| AZ-6B7N | 6.51 | 6.79 | 6.47 |
| AZ-6N7B | 10.79 | - | 10.76 |
| AZ-1B2N | 12.98 | 13.11 | 12.81 |
| AZ-7B8N | 13.15 | 13.18 | - |
| AZ-8N9B | 14.33 | 13.61 | 14.45 |
| AZ-1B9N | 19.36 | 19.37 | 19.25 |
| AZ-9B10N | 19.82 | 20.62 | 19.82 |

**Table S3.** The Cartesian coordinates of BN-doped azulene **1N9B** isomer at basis sets, functionals, and different dispersion methods.

| **1N9B** | **CAM-B3LYP/6-311G (d,p)** |
| --- | --- |
|  | 6 -2.236609000 1.476364000 0.000000000  7 -0.961394000 2.004018000 0.000000000  6 -2.240476000 0.117340000 0.000000000  5 0.000000000 0.930979000 0.000000000  6 -0.879827000 -0.347766000 0.000000000  6 1.495045000 1.028105000 0.000000000  6 -0.465064000 -1.652305000 0.000000000  1 1.976712000 2.004780000 0.000000000  1 -1.226929000 -2.430401000 0.000000000  6 2.348526000 -0.042426000 0.000000000  6 0.868462000 -2.115867000 0.000000000  1 3.414545000 0.176332000 0.000000000  6 2.059122000 -1.430692000 0.000000000  1 -3.129578000 -0.497288000 0.000000000  1 -0.812677000 2.998249000 0.000000000  1 -3.098752000 2.130541000 0.000000000  1 0.969991000 -3.197572000 0.000000000  1 2.941372000 -2.064176000 0.000000000 |
|  | **CAM-B3LYP/6-311+G(d,p)** |
|  | 6 -2.238302000 1.477081000 0.000000000  7 -0.962529000 2.005355000 0.000000000  6 -2.240741000 0.117362000 0.000000000  5 0.000000000 0.930789000 0.000000000  6 -0.879092000 -0.347599000 0.000000000  6 1.495283000 1.028346000 0.000000000  6 -0.465008000 -1.652886000 0.000000000  1 1.976576000 2.005045000 0.000000000  1 -1.227081000 -2.430668000 0.000000000  6 2.349620000 -0.042590000 0.000000000  6 0.868834000 -2.116966000 0.000000000  1 3.415429000 0.176655000 0.000000000  6 2.060193000 -1.431424000 0.000000000  1 -3.130028000 -0.497057000 0.000000000  1 -0.813596000 2.999890000 0.000000000  1 -3.100929000 2.130363000 0.000000000  1 0.970252000 -3.198630000 0.000000000  1 2.942363000 -2.064975000 0.000000000 |
|  | **CAM-B3LYP/cc-pVTZ** |
|  | 6 -2.233293000 1.472260000 0.000000000  7 -0.962209000 2.001338000 0.000000000  6 -2.235577000 0.115990000 0.000000000  5 0.000000000 0.929816000 0.000000000  6 -0.878062000 -0.347920000 0.000000000  6 1.493861000 1.026469000 0.000000000  6 -0.463614000 -1.649319000 0.000000000  1 1.975419000 2.000404000 0.000000000  1 -1.223414000 -2.426239000 0.000000000  6 2.345030000 -0.042230000 0.000000000  6 0.867185000 -2.110951000 0.000000000  1 3.408749000 0.175671000 0.000000000  6 2.055375000 -1.427167000 0.000000000  1 -3.122765000 -0.497440000 0.000000000  1 -0.817238000 2.993712000 0.000000000  1 -3.095332000 2.122752000 0.000000000  1 0.969376000 -3.190202000 0.000000000  1 2.935232000 -2.059895000 0.000000000 |
|  | **CAM-B3LYP/cc-pVQZ** |
|  | 6 -2.231685000 1.473612000 0.000000000  7 -0.960205000 2.000956000 0.000000000  6 -2.235294000 0.117555000 0.000000000  5 0.000000000 0.929269000 0.000000000  6 -0.877836000 -0.347595000 0.000000000  6 1.493304000 1.025522000 0.000000000  6 -0.464493000 -1.649055000 0.000000000  1 1.974533000 1.998918000 0.000000000  1 -1.224261000 -2.425090000 0.000000000  6 2.344225000 -0.043073000 0.000000000  6 0.866054000 -2.111255000 0.000000000  1 3.407317000 0.174536000 0.000000000  6 2.054246000 -1.427927000 0.000000000  1 -3.122608000 -0.494692000 0.000000000  1 -0.813430000 2.992382000 0.000000000  1 -3.092518000 2.124644000 0.000000000  1 0.967889000 -3.189902000 0.000000000  1 2.933390000 -2.060535000 0.000000000 |
|  | **CAM-B3LYP/Def2TZVPP** |
|  | 6 -2.232523000 1.474180000 0.000000000  7 -0.961126000 2.001778000 0.000000000  6 -2.235880000 0.117362000 0.000000000  5 0.000000000 0.929448000 0.000000000  6 -0.878328000 -0.347699000 0.000000000  6 1.493940000 1.025976000 0.000000000  6 -0.464449000 -1.649738000 0.000000000  1 1.975472000 2.000060000 0.000000000  1 -1.224747000 -2.426315000 0.000000000  6 2.345262000 -0.043171000 0.000000000  6 0.866376000 -2.111877000 0.000000000  1 3.409115000 0.174677000 0.000000000  6 2.055171000 -1.428247000 0.000000000  1 -3.123844000 -0.495335000 0.000000000  1 -0.814402000 2.993854000 0.000000000  1 -3.094219000 2.125409000 0.000000000  1 0.968227000 -3.191310000 0.000000000  1 2.934868000 -2.061451000 0.000000000 |
| **1N9B** | **CAM-B3LYP-BJ3/6-311G(d,p)** |
|  | 6 -2.236808000 1.475516000 0.000000000  7 -0.961825000 2.003103000 0.000000000  6 -2.240220000 0.116462000 0.000000000  5 0.000000000 0.930241000 0.000000000  6 -0.879423000 -0.348390000 0.000000000  6 1.494191000 1.028368000 0.000000000  6 -0.464049000 -1.652495000 0.000000000  1 1.976539000 2.004487000 0.000000000  1 -1.224632000 -2.431554000 0.000000000  6 2.347754000 -0.041527000 0.000000000  6 0.869129000 -2.114702000 0.000000000  1 3.413504000 0.177462000 0.000000000  6 2.058901000 -1.429025000 0.000000000  1 -3.129532000 -0.497777000 0.000000000  1 -0.814089000 2.997487000 0.000000000  1 -3.098719000 2.129901000 0.000000000  1 0.971646000 -3.196100000 0.000000000  1 2.941200000 -2.062080000 0.000000000 |
|  | **CAM-B3LYP-GD3/6-311G(d,p)** |
|  | 6 -2.236609000 1.476364000 0.000000000  7 -0.961394000 2.004018000 0.000000000  6 -2.240476000 0.117340000 0.000000000  5 0.000000000 0.930979000 0.000000000  6 -0.879827000 -0.347766000 0.000000000  6 1.495045000 1.028105000 0.000000000  6 -0.465064000 -1.652305000 0.000000000  1 1.976712000 2.004780000 0.000000000  1 -1.226929000 -2.430401000 0.000000000  6 2.348526000 -0.042426000 0.000000000  6 0.868462000 -2.115867000 0.000000000  1 3.414545000 0.176332000 0.000000000  6 2.059122000 -1.430692000 0.000000000  1 -3.129578000 -0.497288000 0.000000000  1 -0.812677000 2.998249000 0.000000000  1 -3.098752000 2.130541000 0.000000000  1 0.969991000 -3.197572000 0.000000000  1 2.941372000 -2.064176000 0.000000000 |
| **1N9B** | **B3LYP/6-311G(d,p)** |
|  | 6 -2.244638000 1.484485000 0.000000000  7 -0.970065000 2.013152000 0.000000000  6 -2.242723000 0.113140000 0.000000000  5 0.000000000 0.934488000 0.000000000  6 -0.884922000 -0.348569000 0.000000000  6 1.494846000 1.033533000 0.000000000  6 -0.462421000 -1.663991000 0.000000000  1 1.979358000 2.010168000 0.000000000  1 -1.222612000 -2.445022000 0.000000000  6 2.357986000 -0.044515000 0.000000000  6 0.869786000 -2.123778000 0.000000000  1 3.424153000 0.177620000 0.000000000  6 2.070420000 -1.430607000 0.000000000  1 -3.134187000 -0.499863000 0.000000000  1 -0.825535000 3.009394000 0.000000000  1 -3.108971000 2.136704000 0.000000000  1 0.975603000 -3.205966000 0.000000000  1 2.952645000 -2.065737000 0.000000000 |
|  | **B97d/6-311G(d,p)** |
|  | 6 -2.259097000 1.485520000 0.000000000  7 -0.985086000 2.022122000 0.000000000  6 -2.247954000 0.102282000 0.000000000  5 0.000000000 0.939543000 0.000000000  6 -0.888740000 -0.352369000 0.000000000  6 1.501019000 1.046393000 0.000000000  6 -0.460077000 -1.675723000 0.000000000  1 1.990529000 2.026804000 0.000000000  1 -1.222667000 -2.461815000 0.000000000  6 2.370355000 -0.039762000 0.000000000  6 0.875952000 -2.131066000 0.000000000  1 3.441431000 0.184216000 0.000000000  6 2.083476000 -1.428676000 0.000000000  1 -3.140723000 -0.518556000 0.000000000  1 -0.850421000 3.023150000 0.000000000  1 -3.130125000 2.137639000 0.000000000  1 0.987658000 -3.217664000 0.000000000  1 2.970317000 -2.065935000 0.000000000 |
|  | **CAM-B3LYP/6-311G(d,p)** |
|  | 6 -2.237204000 1.476245000 0.000000000  7 -0.962148000 2.003926000 0.000000000  6 -2.241195000 0.117283000 0.000000000  5 0.000000000 0.930942000 0.000000000  6 -0.880214000 -0.347983000 0.000000000  6 1.495364000 1.027926000 0.000000000  6 -0.464449000 -1.652481000 0.000000000  1 1.978659000 2.003793000 0.000000000  1 -1.224885000 -2.431926000 0.000000000  6 2.348868000 -0.042431000 0.000000000  6 0.869122000 -2.115370000 0.000000000  1 3.414798000 0.176389000 0.000000000  6 2.059538000 -1.430312000 0.000000000  1 -3.131089000 -0.496322000 0.000000000  1 -0.815028000 2.998527000 0.000000000  1 -3.099085000 2.130886000 0.000000000  1 0.970989000 -3.196991000 0.000000000  1 2.941698000 -2.063821000 0.000000000 |
|  | **HSEH1PBE (HSE06) /6-311G(d,p)** |
|  | 6 -2.234750000 1.482250000 0.000000000  7 -0.966805000 2.008024000 0.000000000  6 -2.236512000 0.113321000 0.000000000  5 0.000000000 0.931688000 0.000000000  6 -0.884049000 -0.347603000 0.000000000  6 1.493419000 1.031936000 0.000000000  6 -0.463727000 -1.660593000 0.000000000  1 1.979107000 2.008402000 0.000000000  1 -1.225112000 -2.441173000 0.000000000  6 2.352228000 -0.045295000 0.000000000  6 0.865123000 -2.118541000 0.000000000  1 3.419421000 0.174778000 0.000000000  6 2.063026000 -1.427354000 0.000000000  1 -3.129568000 -0.498349000 0.000000000  1 -0.821911000 3.002575000 0.000000000  1 -3.099336000 2.135660000 0.000000000  1 0.971654000 -3.201228000 0.000000000  1 2.944839000 -2.064008000 0.000000000 |
|  | **MN12/6-311G(d,p)** |
|  | 6 -2.229736000 1.485605000 0.000000000  7 -0.960108000 2.006591000 0.000000000  6 -2.235385000 0.118004000 0.000000000  5 0.000000000 0.931962000 0.000000000  6 -0.882902000 -0.344983000 0.000000000  6 1.491983000 1.028600000 0.000000000  6 -0.467767000 -1.659466000 0.000000000  1 1.981121000 2.007967000 0.000000000  1 -1.233660000 -2.441625000 0.000000000  6 2.350403000 -0.048002000 0.000000000  6 0.860767000 -2.119934000 0.000000000  1 3.422322000 0.169675000 0.000000000  6 2.058796000 -1.430440000 0.000000000  1 -3.132393000 -0.492506000 0.000000000  1 -0.809756000 3.004886000 0.000000000  1 -3.093273000 2.146488000 0.000000000  1 0.966588000 -3.206616000 0.000000000  1 2.942850000 -2.070524000 0.000000000 |
|  | **MN15/6-311G(d,p)** |
|  | 6 -2.238843000 1.482109000 0.000000000  7 -0.963877000 2.011362000 0.000000000  6 -2.242289000 0.116614000 0.000000000  5 0.000000000 0.932597000 0.000000000  6 -0.882156000 -0.346313000 0.000000000  6 1.497402000 1.032254000 0.000000000  6 -0.467476000 -1.660035000 0.000000000  1 1.981556000 2.008919000 0.000000000  1 -1.231904000 -2.437057000 0.000000000  6 2.354929000 -0.044703000 0.000000000  6 0.866664000 -2.123191000 0.000000000  1 3.421997000 0.173024000 0.000000000  6 2.063204000 -1.433207000 0.000000000  1 -3.132049000 -0.498797000 0.000000000  1 -0.815021000 3.008521000 0.000000000  1 -3.102105000 2.135999000 0.000000000  1 0.970989000 -3.205485000 0.000000000  1 2.945080000 -2.068816000 0.000000000 |
|  | **PBE0DH/6-311G(d,p)** |
|  | 6 -2.228438000 1.479496000 0.000000000  7 -0.962093000 2.003571000 0.000000000  6 -2.233752000 0.115230000 0.000000000  5 0.000000000 0.929898000 0.000000000  6 -0.881921000 -0.346181000 0.000000000  6 1.492938000 1.029534000 0.000000000  6 -0.465672000 -1.656095000 0.000000000  1 1.979048000 2.002316000 0.000000000  1 -1.225791000 -2.433562000 0.000000000  6 2.347562000 -0.045778000 0.000000000  6 0.862226000 -2.114732000 0.000000000  1 3.412059000 0.171898000 0.000000000  6 2.056747000 -1.426754000 0.000000000  1 -3.125224000 -0.493547000 0.000000000  1 -0.817001000 2.994731000 0.000000000  1 -3.089845000 2.132599000 0.000000000  1 0.968066000 -3.194430000 0.000000000  1 2.935196000 -2.062819000 0.000000000 |
|  | **wB97XD/6-311G(d,p)** |
|  | 6 -2.237149000 1.479199000 0.000000000  7 -0.962811000 2.007010000 0.000000000  6 -2.243518000 0.118001000 0.000000000  5 0.000000000 0.932809000 0.000000000  6 -0.882097000 -0.348403000 0.000000000  6 1.499590000 1.030300000 0.000000000  6 -0.467369000 -1.655739000 0.000000000  1 1.985098000 2.006127000 0.000000000  1 -1.228996000 -2.435287000 0.000000000  6 2.352520000 -0.043781000 0.000000000  6 0.867723000 -2.118608000 0.000000000  1 3.419868000 0.173203000 0.000000000  6 2.060526000 -1.432625000 0.000000000  1 -3.135386000 -0.493789000 0.000000000  1 -0.815931000 3.000666000 0.000000000  1 -3.099007000 2.134991000 0.000000000  1 0.970013000 -3.201175000 0.000000000  1 2.942664000 -2.067909000 0.000000000 |

**Table S4:** Computed absorption spectra (λ_max_, nm/eV) of azulene and BN-doped azulene at TD-DFT(PCM) TD-CAM-B3LYP/6-31+G(d,p)// B3LYP/6-31+G(d,p)in acetone.

| Compound | absorption energy | | *F* | Transitions |
| --- | --- | --- | --- | --- |
|  | (nm) | (eV) |  |  |
| AZ | 257 | 4.83 | 1.3292 | H-1→L (54%), H→L+1 (44%) |
| AZ-1N2B | 264 | 4.70 | 0.4064 | H-1→L (71%), H→L+1 (14%) |
| AZ-1B2N | 235 | 5.27 | 0.3738 | H-1→L (43%), H→L+5 (41%) |
| AZ-1N9B | 201 | 6.16 | 0.3865 | H-2→L (78%) |
| AZ-1B9N | 261 | 4.76 | 0.8273 | H-1→L (60%), H→L+1 (14%) |
| AZ-6N7B | 248 | 5.00 | 0.8799 | H-1→L (48%), H→L+1 (38%), |
| AZ-6B7N | 253 | 4.90 | 1.0738 | H-1→L (42%), H-1→L+1 (31%), H→L+1 (20%) |
| AZ-7N8B | 234 | 5.29 | 0.6149 | H-1→L+1 (89%) |
| AZ-7B8N | 259 | 4.78 | 0.7867 | H-1→L (19%), H→L+1 (77%) |
| AZ-8N9B | 229 | 5.40 | 0.5649 | H-1→L+1 (90%) |
| AZ-8B9N | 236 | 5.25 | 0.5897 | H-1→L (79%),H→L+1 (17%) |
| AZ-9B10N | 280 | 4.43 | 0.5081 | H-1→L (63%), H→L+1 (31%) |

**Table S5.** The calculated and experimental wavelength of S_0_-S_1_ and S_0_-S_2_ transitions for azulene ^a^

| **Level of calculation** | **S_0_-S_1_/nm** | **S_0_-S_2_/nm** |
| --- | --- | --- |
| B3LYP/6-31+G(d,p) (gas) | 523 (0.0073) | 344 (0.0032) |
| B3LYP/6-31+G(d,p) (acetone) | 516 (0.0107) | 342 (0.0058) |
| CAM-B3LYP/6-31+G(d,p) (acetone) | 507 (0.012) | 326 (0.0066) |
| Exp. (hexanes) [1] | 580 (0.0348) | 341(0.0401) |
| Exp. (ethanol) [1] | 576 (0.0362) | 340 (0.0420) |
| TD-PBE0/6-31G(d) [2] | 502 (0.008) | 329 (0.002) |

^a^Values in parentheses represent oscillator strength

[1] S. V Shevyakov, H. Li, R. Muthyala, A.E. Asato, J.C. Croney, D.M. Jameson, R.S.H. Liu, J. Phys. Chem. A. 107 (2003) 3295–3299. The experimental absorption spectra are performed in hexane and ethanol.

[2] K. Veys, D. Escudero, J. Phys. Chem. A 124.36 (2020): 7228-7237.

a)


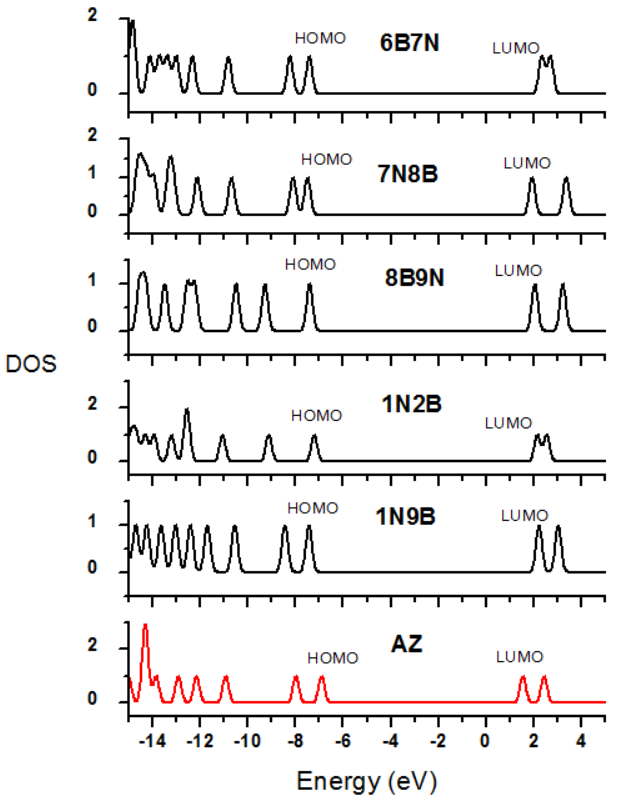


Figure S1. The Density of states (DOS) for a) the most stable BN-doped azulene and b) less stable BN-doped isomers at the G3MP2 level.

b)


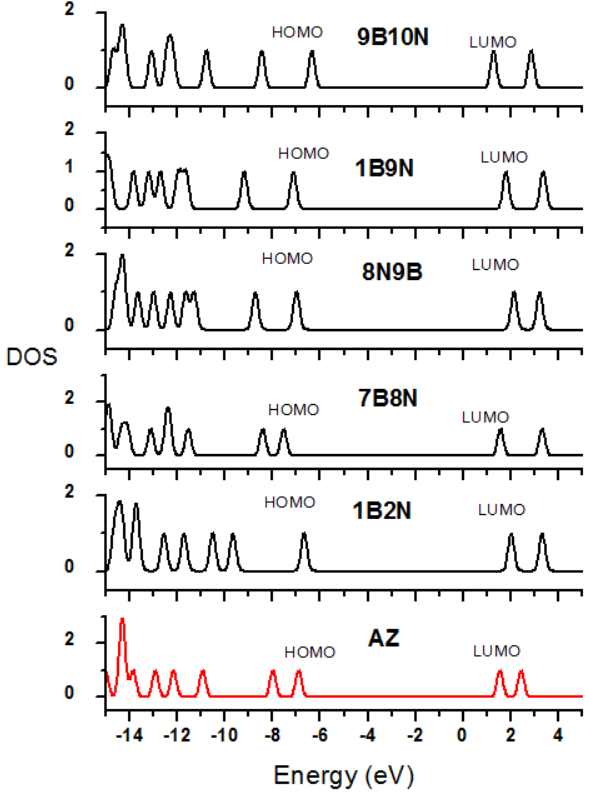


Figure S1. Continued.

**
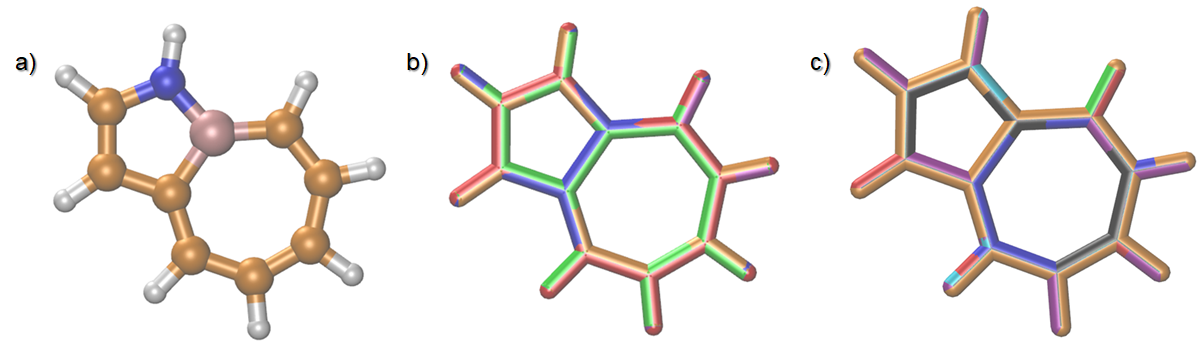
**

Figure S2: The effect of different dispersion corrections; GD3 and GD3BJ (a), basis sets (b), and different functionals (c) on the optimized geometries of the AZ-1N9B isomer. In a, the structure optimized using GD3 corrction is represented in sticks and the one optimized using BJ3 is shown in balls and sticks. The color code for the structures optimized at different basis sets; blue:6-311G(d,p), brown:6-311+G(d,p), green:cc-pVTZ, red:cc-pVQZ, and purple:Def2TZVPP. The color code for the different DFT functionals used in the optimizations benchmark is; blue:CAM-B3LYP, magenta:B3LYP, brown:wB97XD, green: HSEH1PBE (HSE06), red:PBE0DH, purple:MN15, cyan:MN12-SX, and black:B97D.

| 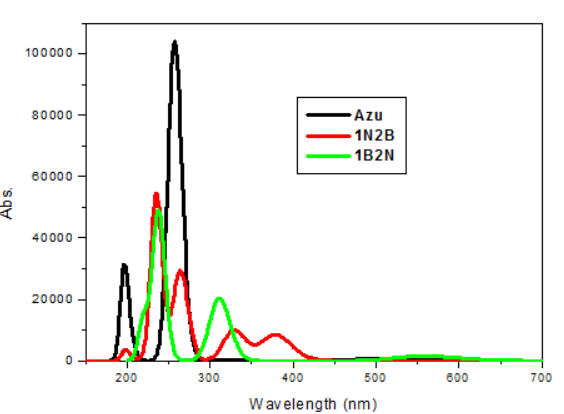 | 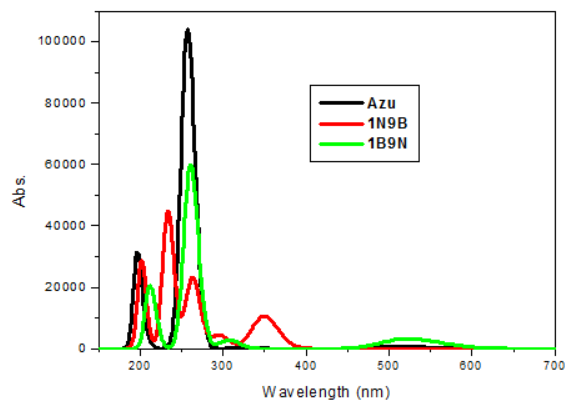 |
| --- | --- |
| 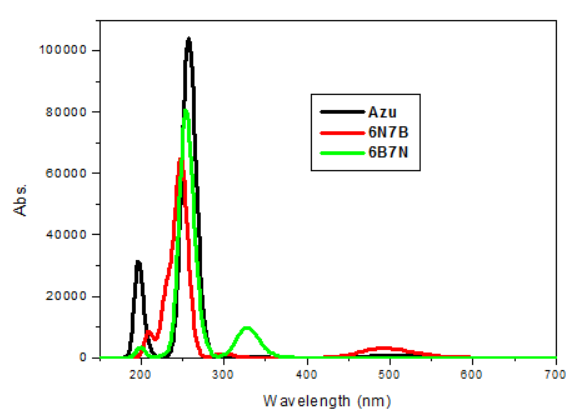 | 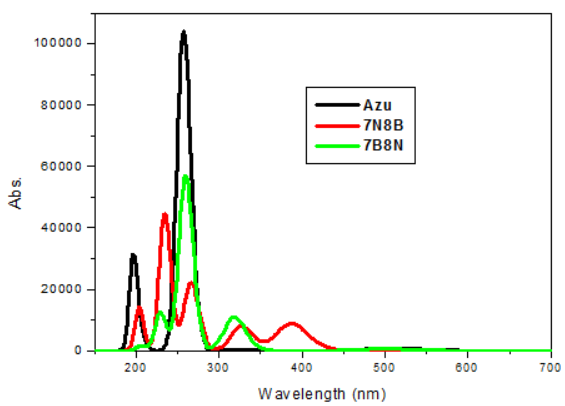 |
|  | 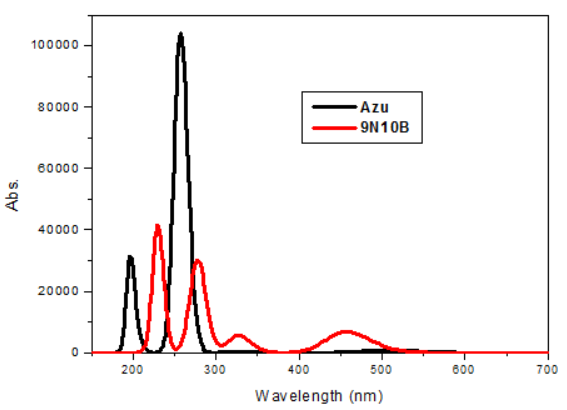 |

Fig.S3. UV-vis spectra for azulene and BN-doped azulene at TD-CAM-B3LYP/6-31+G(d,p)//B3LYP/6-31G(d,p) in acetone.
